# Sodium‐Glucose Cotransporter 2 Inhibitors for Mesenchymal–Epithelial Transition Inhibitor‐Induced Edema

**DOI:** 10.1111/1759-7714.15509

**Published:** 2024-12-13

**Authors:** Takuya Oyakawa, Keita Miura, Nao Muraoka, Kei Iida, Ayano Fujita, Tateaki Naito, Toshiaki Takahashi

**Affiliations:** ^1^ Division of Cardio‐oncology Shizuoka Cancer Center Shizuoka Japan; ^2^ Division of Thoracic oncology Shizuoka Cancer Center Shizuoka Japan; ^3^ Mishimatoukai Hospital Shizuoka Japan

**Keywords:** cardio‐oncology, edema, lung cancer, MET, SGLT2 inhibitor

## Abstract

The effects of sodium‐glucose cotransporter 2 (SGLT2) inhibitors on mesenchymal‐epithelial transition factor (MET) inhibitor‐induced edema remain unclear. In a patient with tepotinib‐induced edema and an N‐terminal pro‐brain natriuretic peptide (NTproBNP) level ≥ 300 pg/mL, the addition of empagliflozin to loop diuretics reduced the edema. This suggests that empagliflozin may be a treatment option for MET inhibitor‐induced edema.

## Graphical abstract

The effects of sodium‐glucose cotransporter 2 (SGLT2) inhibitors on mesenchymal–epithelial transition factor (MET) inhibitor‐induced edema remain unclear. In a patient with tepotinib‐induced edema, the addition of empagliflozin to loop diuretics reduced the edema. This suggests that empagliflozin may be a treatment option for MET inhibitor‐induced edema.

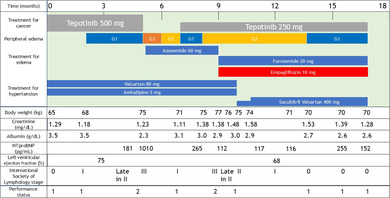

## 1 Introduction

Mesenchymal–epithelial transition factor (MET) inhibitors are used in patients with MET exon 14 skipping mutation‐positive lung cancer. Edema is a common and problematic adverse event associated with MET inhibitors [1]. Furosemide alone has shown limited efficacy in managing this edema [2], and additional therapeutic options are often necessary to maintain cancer treatment.

Sodium‐glucose cotransporter 2 (SGLT2) inhibitors, initially approved for diabetes, have demonstrated effectiveness in treating heart failure. Both dapagliflozin and empagliflozin are approved for heart failure treatment, including in patients with preserved left ventricular ejection fraction [3, 4]. A clinical trial of empagliflozin for heart failure with preserved ejection fraction in patients with NTproBNP levels ≥ 300 pg/mL and limited physical activity demonstrated a significant reduction in heart failure recurrence [4]. SGLT2 inhibitors also increase sodium excretion, promote diuresis, and reduce edema, suggesting potential efficacy for MET inhibitor‐induced edema [5]. Herein, we present a case where empagliflozin was used in a patient with MET inhibitor‐induced edema and an NTproBNP level ≥ 300 pg/mL [4].

### 1.1 Case Report

A 74‐year‐old man, weighing 65 kg, was treated with tepotinib 500 mg for Stage IV MET exon 14 skipping mutation‐positive lung adenocarcinoma as a first‐line therapy. He had no history of cardiac disease. Three months after initiating treatment, the patient developed Grade 1 edema (as per the Common Terminology Criteria for Adverse Events [CTCAE] version 5.0). Five months into the treatment, the edema progressed to Grade 3, prompting the discontinuation of tepotinib and initiation of azosemide 60 mg. Two months later, the edema improved to Grade 1, allowing the resumption of tepotinib at a reduced dose of 250 mg. However, 1 month after reintroducing tepotinib, the edema worsened to Grade 2, and by 2 months, the patient's weight had increased to 77 kg, with worsening edema.

Further intervention was required to continue cancer treatment. The patient's left ventricular ejection fraction was 75%, but NTproBNP was elevated to 1010 pg/mL. Tepotinib was continued at 250 mg, and furosemide 20 mg replaced azosemide. In addition, empagliflozin 10 mg was administered. One week later, the patient experienced reduced edema in the lower and upper limbs. Three weeks into empagliflozin treatment, the patient reported significant improvement in lower limb swelling, with a weight reduction to 75 kg. Given the patient's concurrent hypertension, treated with amlodipine 5 mg and valsartan 80 mg, the therapy was modified to sacubitril/valsartan 200 mg, anticipating further improvement in the edema.

**FIGURE 1:**
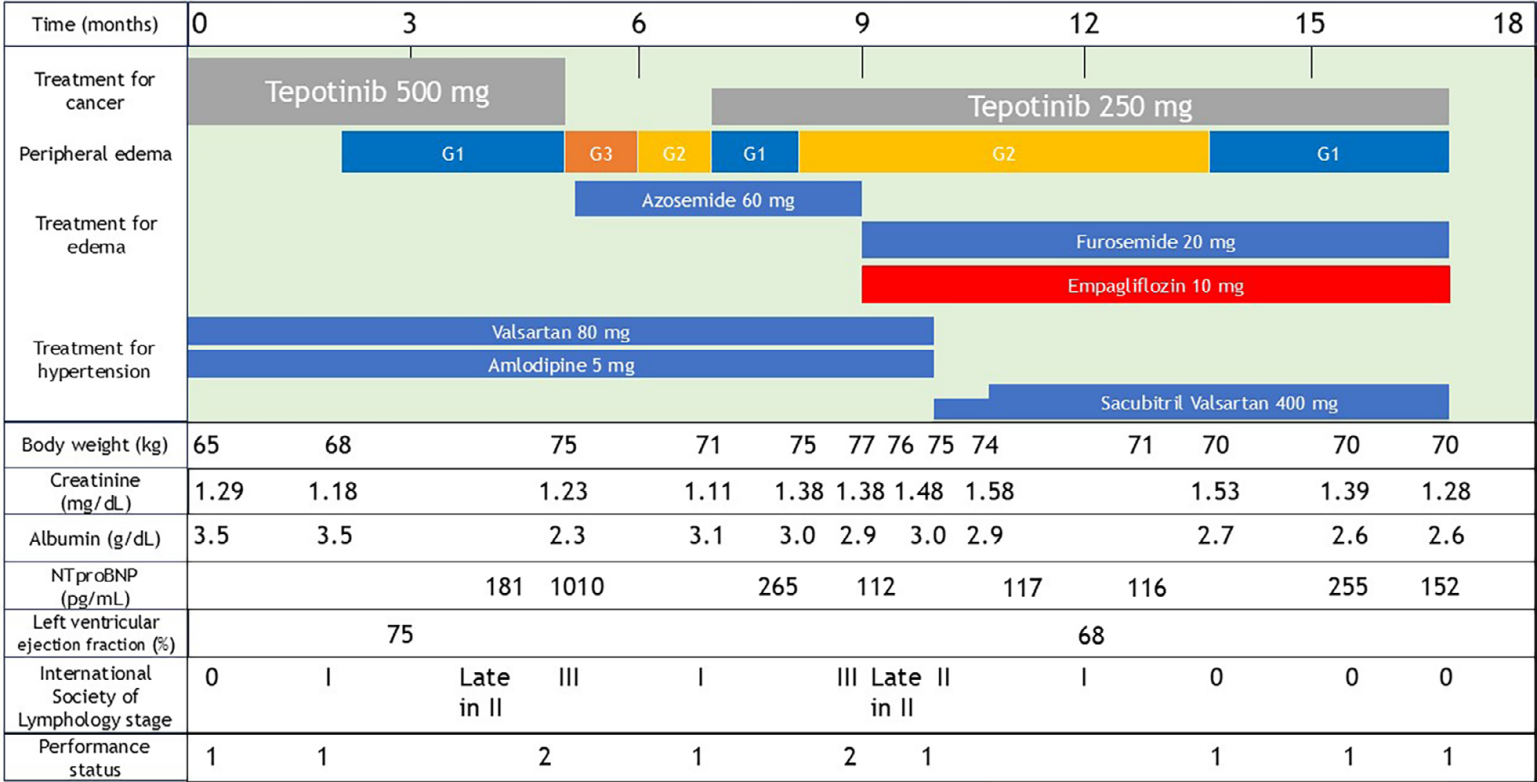
Clinical course of the patient.

Six weeks after initiating empagliflozin, the patient's edema further improved, allowing him to wear his usual shoes again, and his weight dropped to 74 kg. The sacubitril/valsartan dose was increased to 400 mg. Five months into empagliflozin treatment, the patient's edema improved to Grade 1, and it no longer interfered with daily life, and his weight was 70 kg. Tepotinib 250 mg was continued throughout (Figure 1).

The International Society of Lymphology staging system was used to assess edema [6].

## 2 Discussion

We report the first case, to our knowledge, of empagliflozin being used to treat MET inhibitor‐induced edema. In this case, the addition of empagliflozin to loop diuretics led to a noticeable improvement in edema, allowing the patient to continue tepotinib treatment. Empagliflozin was well tolerated, with no adverse events reported. These results suggest that empagliflozin may be a viable option for treating MET inhibitor‐induced edema.

Edema occurs in approximately 70% of patients treated with MET inhibitors, with Grade 3 or higher edema occurring in 9% of cases [2]. Although not life‐threatening, edema can interfere with treatment adherence, making supportive care essential for ongoing cancer therapy. Loop diuretics, such as furosemide, reduce edema by shifting fluid from the interstitial space into blood vessels. In contrast, SGLT2 inhibitors like empagliflozin draw fluid directly from the interstitium, offering a more effective reduction in interstitial volume compared to furosemide [7].

The mechanism by which SGLT2 inhibitors reduce edema is not fully understood; however it may involve inhibition of proton exchange on cell surfaces, which reduces intracellular sodium levels [8]. SGLT2 inhibitors reduce sodium levels in the skin, rather than in muscles, which may explain their effectiveness in treating edema [9].

Another cause of MET inhibitor‐induced edema is hypoalbuminemia [2]. SGLT2 inhibitors have been shown to be effective in treating edema caused by nephrotic syndrome, which is often associated with hypoalbuminemia [10, 11]. Therefore, it is plausible that SGLT2 inhibitors could also alleviate edema related to hypoalbuminemia induced by MET inhibitors.

This study has limitations. As this is a retrospective case report, quantitative evaluation of edema improvement was not possible. Additionally, after the reduction in edema with empagliflozin, the patient was also treated with sacubitril/valsartan, which may have contributed to the observed improvement due to its diuretic effect [12]. Future studies with larger sample sizes and clinical trials are necessary to further validate the efficacy of SGLT2 inhibitors for MET inhibitor‐induced edema.

To date, we have used SGLT2 inhibitors to treat MET inhibitor‐induced edema in three consecutive patients, all of whom have seen a reduction in edema, although to varying degrees.

In conclusion, empagliflozin, when added to standard treatments such as furosemide, may offer a treatment option for managing MET inhibitor‐induced edema.

## Author Contributions

**Takuya Oyakawa:** conceptualization, investigation, methodology, writing – original draft preparation. **Keita Miura:** investigation, writing – review and editing. **Nao Muraoka:** writing – review and editing. **Kei Iida:** writing – review and editing. **Ayano Fujita:** writing – review and editing. **Tateaki Naito:** writing – review and editing. **Toshiaki Takahashi:** writing – review and editing.

## Ethics Statement

This study was approved by our institutional review board (J2024‐87‐2024‐1‐3). All procedures involving human participants performed in this study were in accordance with the ethical standards of the institutional and/or national research committee and with the 1964 Declaration of Helsinki and its later amendments and comparable ethical standards.

## Consent

Written informed consent was obtained from all patients.

## Conflicts of Interest

Takuya Oyakawa received honoraria from Pfizer, Daiichi Sankyo, TOA EIYO, Novartis, AstraZeneca, and Bristol‐Myers Squibb outside the submitted work. Keita Miura received honoraria from AstraZeneca K.K., Chugai Pharmaceutical Co Ltd., and MSD K.K. outside the submitted work. The other authors declare no conflicts of interest.

## Data Availability

The data that support the findings of this study are available on request from the corresponding author. The data are not publicly available due to privacy or ethical restrictions.
